# A Strong Correlation Between the Severity of Flatfoot and Symptoms of Knee Osteoarthritis in 95 Patients

**DOI:** 10.3389/fsurg.2022.936720

**Published:** 2022-06-30

**Authors:** Min Zhang, Mao-dan Nie, Xin-zheng Qi, Song Ke, Jun-wei Li, Yang-yang Shui, Zhuo-yue Zhang, Min Wang, Cheng-Kung Cheng

**Affiliations:** ^1^ Advanced Innovation Center for Biomedical Engineering, School of Biological Science and Medical Engineering, Beihang University, Beijing, China; ^2^ School of Biomedical Engineering, Shanghai Jiao Tong University, Shanghai, China; ^3^ Department of Orthopedics, Xinqiao Hospital, Third Military Medical University, Chongqing, China

**Keywords:** knee osteoarthritis, flatfoot, K-L grade, FTA, knee pain, knee function, knee stiffness

## Abstract

**Objective:**

The purpose of this study is to assess the association between the presence and severity of flatfoot and symptoms of knee OA.

**Methods:**

95 participants with knee OA were recruited from a patient cohort at a regional hospital. Symptoms of knee OA, including knee degeneration, femorotibial alignment, pain, stiffness and dysfunction were assessed using the Kellgren-Lawrence (K-L) grading system, femoral-tibial angle (FTA), and Western Ontario and McMaster Universities Osteoarthritis Index (WOMAC). Participants were divided into groups with flatfoot (mild, moderate and severe) and without flatfoot based on the Clarke's angle. Linear regression and ordinal logistic regression were used for statistical analysis, as appropriate.

**Results:**

Having flatfoot was associated with a significantly increased risk of having a higher K-L grade (OR: 20.03; 95% CI, 5.88, 68.27; *p* < 0.001), smaller FTA (Beta: −2.96; 95% CI, −4.41, −1.50; *p* < 0.001), higher pain score (Beta: 0.47; 95% CI, 0.24, 0.69; *p* < 0.001) and greater loss of function (Beta: 0.25; 95% CI, 0.02, 0.48; *p* = 0.03). Severe grades of flat feet were associated with a higher K-L grade (OR: 0.19; 95% CI, 0.08, 0.44; *p* < 0.001), smaller FTA (Beta: 1.51; 95% CI, 0.66, 2.35; *p* = 0.001), higher pain score (Beta: −0.25; 95% CI, −0.39, −0.11; *p* = 0.001), greater stiffness (Beta: −0.24; 95% CI, −0.38, −0.09; *p* = 0.002) and greater loss of function (Beta: −0.27; 95% CI, −0.41, −0.14; *p* < 0.001).

**Conclusion:**

The results indicated that the severity of flattening is significantly associated with symptoms of knee OA. For the conservative management of knee OA, both flatfoot and its severity should be carefully considered.

## Introduction

Knee osteoarthritis (OA) is a debilitating joint disease that results in structural damage to articular cartilage and bone. Abnormal mechanical loading of lower limbs has been reported to be associated with symptoms of knee OA (1). The foot plays an important role in sculpting the pattern of postural alignment throughout the lower extremity, absorbing ground reaction forces, and maintaining normal joint motion (2). The motion of the foot and knee are coupled in a closed kinematic chain during weight-bearing activities, which can cause excessive knee rotation in people with flatfoot (3). Due to this interdependent relationship, abnormal foot posture can lead to some knee pathologies, such as knee OA.

People with flatfoot often complain of knee pain (4) and suffer knee degeneration (5). Studies have shown that knee pain in flatfooted people with knee OA can be relieved by taking steps to correct the flatfoot (6). Despite the known biomechanical link between flatfoot and mechanical stress in the knee, previous studies only considered the relationship between the presence of flatfoot and knee pain or knee degeneration (3, 5, 7). The association between the severity of flatfoot and knee OA is still unclear. In addition to knee pain and degeneration (8), knee malalignment (9), stiffness, and dysfunction (10) are also key factors for physicians when diagnosing knee OA. Thus, assessing the relationship between the characteristics of the knee and foot can help with accurate diagnosis and with devising a suitable correction plan.

This study aimed to systematically assess the correlation between the severity of flatfoot and the symptoms of knee OA, including knee degeneration, lower limb alignment, knee pain, stiffness, and function. This study hypothesized that not only the presence of flatfoot but also the severity of flatfoot is associated with the stated knee symptoms.

## Materials and Methods

### Study Population

Patients were recruited from the orthopedic clinic of a well-known orthopaedic hospital in Chongqing, China. Invitation letters were issued to all patients (*n* = 146) diagnosed in August 2019 as having knee osteoarthritis according to the American College of Rheumatology Clinical Criteria. The inclusion criteria were an ability to walk independently, the presence of knee pain, no severe foot deformity other than flatfoot, and no foot lesions. Exclusion criteria were a history of knee injury or surgery, rheumatoid osteoarthritis, and mental disorders. A total of 120 patients provided positive responses, but insufficient data were available for 25 of these patients. Of the 95 patients included in the study, 84 patients exhibited bilateral knee OA, and 11 patients suffered unilateral knee OA. To minimize bias, only one knee from each patient was selected. For patients with bilateral knee OA, the more painful knee was evaluated (10). Characteristics of all patients are shown in Table 1.

**Table 1:** Demographic characteristics, participant-level characteristics, and foot posture of the research population.

Subject characteristics	
Age (years)	58.0 ± 10.5
Male, *N* (%) / Female, *N* (%)	14 (14.7) / 81 (85.3)
BMI (kg/m²), Median (IQR)	24.97 (23.31, 27.70)
Participant-level characteristics	
Severity of knee OA (K-L grade), *N* (%)	
K-L 2	28 (29.5)
K-L 3	51 (53.7)
K-L 4	16 (16.8)
FTA (°), Median (IQR)	−7.35 (−9.12, −6.02)
Pain (WOMAC score), Median (IQR)	1.94 (1.60, 2.28)
Stiffness (WOMAC score), Median (IQR)	1.40 (0.80, 1.60)
Physical function (WOMAC score), Mean **±** SD	1.73 ± 0.49
Foot posture (Clarke's angle), (°)	28.47 ± 13.24

### Foot Posture Assessment

**Figure 1:**
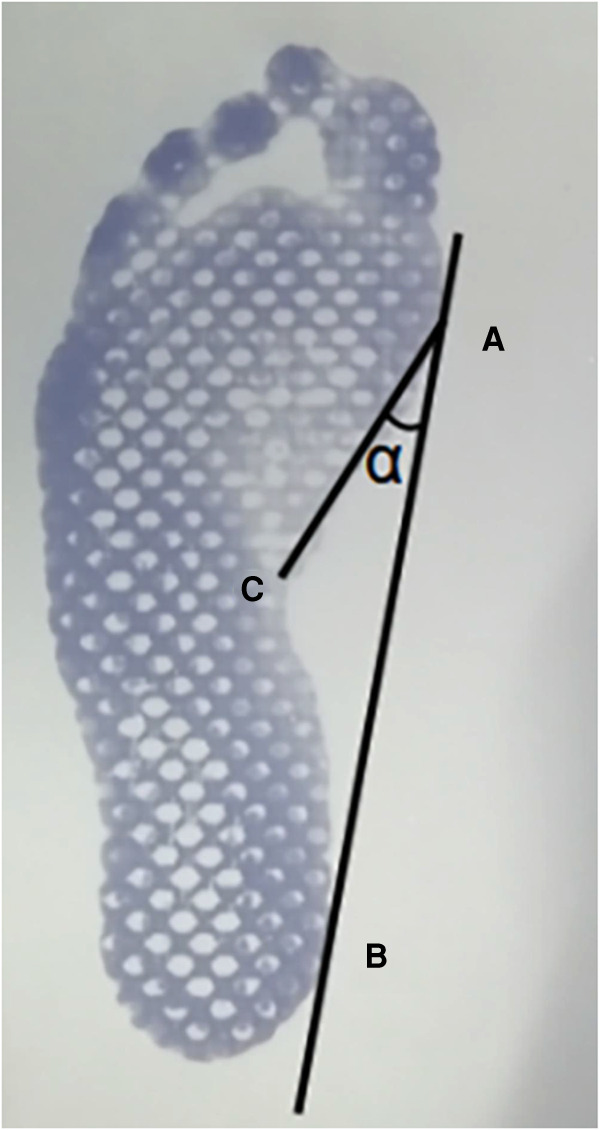
Clarke's angle measurement method using footprints. (**A**) the most medial point of the sole; (**B**) the most medial point of the heel; (**C**) the most concave point on the foot profile; *α*: the Clarke's angle.

Footprints have been widely used in the diagnosis of flatfoot and could draw a consistent conclusion with that by using radiological data, which is an effective method to measure the degree of arch collapse and diagnose flatfoot (11, 12). Static footprints were obtained by having each patient stand on a foot printer (Mingscan Footprinter, Vers Tech Science Co. Ltd., Taiwan) in a relaxed bipedal stance with their toes pointed forward. The Clarke's angle (CA) (*α*) referred to the angle formed by the intersection of line ab and line ac (14) on a footprint (Figure 1) and was used to diagnose flatfoot in this study. The CA was measured three times in each footprint by using PicPick (version 5.0.7, Korea) (accuracy: 0.01°), and the average value was taken as the value for that foot. Clarke's angle is not only highly accurate in diagnosing flatfoot, but also widely used in the assessment of the severity of flatfoot (13–15). Patients in this study were characterized as having flatfoot when CA ≤ 42°. The severity of the flatfoot was classified as mild if 42° ≥ CA > 35°, moderate if 35° ≥ CA > 30°, or severe if 30° ≥ CA (16). The intra-interclass correlation coefficient of CA was 0.993, indicating good reliability (17).

### Evaluation of OA-Related Knee Symptoms

**Figure 2:**
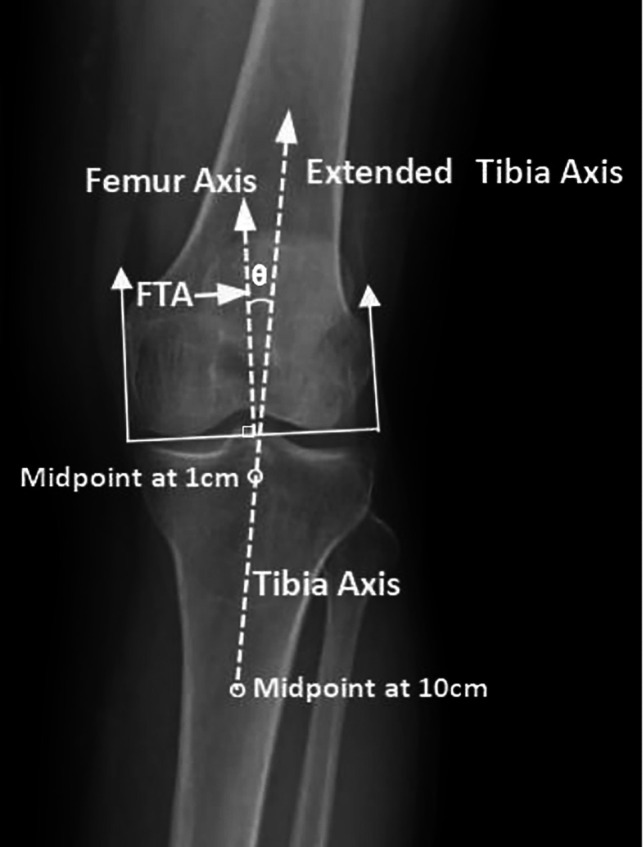
Measurement of femorotibial alignment with a short knee radiograph.

Each patient was invited to have a weight-bearing anteroposterior radiograph with their legs fully extended and the patella facing forward. The same machine (uDR 770i, United Imaging Healthcare Co., Ltd., China) in our medical imaging department was used for all patients by the same operator. Knee OA symptoms including knee degeneration, knee malalignment, knee dysfunction, stiffness, and knee pain were assessed. The K-L grading system was used to evaluate knee degeneration based on subchondral bone sclerosis, osteophytes, articular cartilage, and other symptoms using a scale of 0 to 4 (18). Knee malalignment was assessed by the femoral-tibial angle (FTA) using the method recommended by Moyer et al. (19). The FTA (*θ*) was measured as the angle formed by the intersection of the femoral and tibial axes using PicPick software (accuracy: 0.01°) (Figure 2). The femoral axis was plotted perpendicular to a line tangent to the base of the femoral condyles, and the tibial axis connected the centers of the tibial shaft at points 1 cm and 10 cm distal to the tibial plateau. The FTA of each knee was measured three times, with the average being considered as the value for that knee. The Western Ontario and McMaster Universities Osteoarthritis Index (WOMAC) scale is often used in clinical settings to assess the severity of knee OA regarding knee physical function, stiffness, and pain (20). This questionnaire assesses 3 parameters (physical function, stiffness, and pain) that include 24 separate items for consideration. Each item follows a 5-point Likert rating scale with a range of 0 to 4. The score for each parameter was calculated as the sum of scores of all constituent items divided by the number of items assessed, with a higher score being considered more severe. The intra-interclass correlation coefficient of the K-L grade, FTA, knee function, stiffness, and pain were all over 0.9, indicating good reliability (17).

### Statistical Analysis

Two assessments were performed in this study. In the first assessment, the patients were divided into with flatfoot group and without flatfoot group. The aim was to investigate the association between the presence of flatfoot and the knee OA symptoms (knee degeneration, femorotibial alignment, knee pain, stiffness, and dysfunction). In the other assessment, the patients with flatfoot were further classified as having mild, moderate, or severe flatfoot. This assessment aimed to investigate the association between the severity of flatfoot and knee OA symptoms (knee degeneration, femorotibial alignment, knee pain, stiffness, and dysfunction). Sample sizes were assessed using *a priori* power analysis with a significance level of 0.05 (type - I error) and an expected power of 0.9 (21). K-L was a categorical variable, and thus the comparison of K-L grade between groups (with or without flatfoot) was performed using a Chi-square test. Determining a suitable statistical tool for continuous variables, such as FTA, knee pain score, knee stiffness, and function, depends on the normality of the data. The physical functional scores were found to conform to a normal distribution, but the FTA, knee pain scores, and stiffness did not. Thus, the difference in function between the patients with and without flatfoot was compared using a t-test for normal continuous variables, and the differences in FTA, pain scores, and stiffness were compared using a Mann-Whitney U test for non-parametric continuous variables. The assessment of the severity of flatfoot (mild, moderate, or severe) showed that FTA conformed to a normal distribution, but the pain, stiffness, and physical function scores did not. Therefore, the difference in FTA was compared using a one-way ANOVA test, and differences in pain, stiffness, and physical function were compared by a Kruskal-Wallis test.

Linear regression analysis (10) was used to assess how the presence (with or without flatfoot) and severity (mild, moderate, and severe) of flatfoot affect the continuous dependent variables FTA, knee pain, stiffness, and function. An ordinal logistic regression analysis was used to evaluate how the presence or severity affects ordered dependent variables, which was the K-L grade in this study. The parameters of knee OA were assigned as dependent variables and the presence and severity of flatfoot were designated as independent variables in the regression analyses. Because the relationship to gender, age, and BMI were widely reported for both knee OA and flatfoot (22, 23), these parameters were used as covariates to adjust the regression model (10) to reduce the effect on the outcome. All statistical analyses were performed using SPSS 22.0 (SPSS, IBM, USA) with a significance level of *p* < 0.05.

### Ethics and Potential Conflicts of Interests

Approval was obtained from the institutional medical ethics committee of Beihang University biological science and medical engineering review board (No.: BM20200096). All patients provided written informed consent prior to participation after being briefed on the study’s purpose and protocol. The dataset that is necessary to replicate the main findings can be obtained from the authors upon reasonable request.

## Results

**Figure 3:**
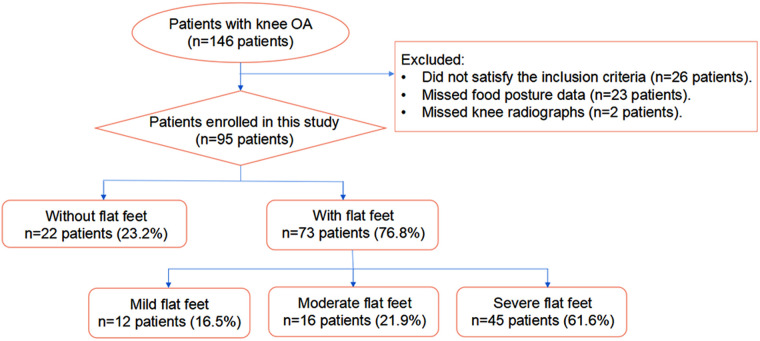
Flow chart showing the distribution of study participants.

Figure 3 shows the characteristics of patients included in this study. 73 of the 95 patients with knee OA also had ipsilateral flatfoot. Among those, 12 had mild flatfoot, 16 had moderate flatfoot and 45 had severe flatfoot.

In comparison to the subjects without flatfoot, the subjects with ipsilateral flatfoot had a smaller FTA (−7.77 (IQR: −9.88, −6.28) vs. −6.35 (IQR: −7.28, −4.24), (*p *< 0.001)), higher knee pain score (1.97 (IQR: 1.78, 2.28) vs. 1.60 (IQR: 1.28, 1.77), *p *< 0.001), more severe dysfunction (1.97 (IQR: 1.78, 2.28) vs. 1.60 (IQR: 1.28, 1.77), *p* < 0.001) and more severe knee degeneration (K-L) (*p* < 0.001) (Table 2). However, there were no statistical differences in the knee stiffness between the subjects with flatfoot and those without flatfoot.

**Table 2:** Participant -level characteristics of the research knees based on the presence of flatfoot (*n* = 95 patients).

Variables	Without flatfoot (*n* = 22 knees)	With flatfoot *n* = 73 knees)	*p*-value*
Subject characteristics			
Age (years)	60.50 ± 9.054	56.99 ± 10.98	0.175
Male, *N* (%) / Female, *N* (%)	**7 (31.8) / 15 (68.2)**	**7 (9.6) / 66 (90.4)**	**0**.**010**
BMI (kg/m²), Median (IQR)	25.95 (23.86, 27.40)	24.97 (23.44, 27.60)	0.533
Severity of knee OA (K-L grade), *N* (%)	** **	** **	**<0**.**001**
K-L 2	**16(72.7)**	**12** **(16.5)**	** **
K-L 3	**6 (27.3)**	**45** **(61.6)**	** **
K-L 4	**0**	**16** **(21.9)**	** **
FTA (°), Median (IQR)	−**6.35 (**−**7.28,**−**4.24)**	−**7.77** −**(9.88,**−**6.28)**	**<0**.**001**
Pain (WOMAC score), Median (IQR)	**1.60 (1.28,1.77)**	**1.97** **(1.78,2.28)**	**<0**.**001**
Stiffness (WOMAC score), Median (IQR)	1.20 (0.80,1.80)	1.46 (1.10,1.60)	0.653
Physical function (WOMAC score), Mean **±** SD	**1.54 ± 0.43**	**1.79 ± 0.49**	**0**.**033**

*Values from One-way ANOVA test for categorical variables presented as N (%), t-test for normal continuous variables presented as mean ± SD, and Mann-Whitney U Test for variables presented as median (IQR)*.

* *p –Bold values represent statistically significant results*.

This study also compared differences between mild, moderate and severe flatfoot and found significant differences in KL grade (*p* < 0.001), FTA (*p* = 0.003), pain score (*p* = 0.002), knee stiffness (*p* = 0.007) and physical function (*p* = 0.001), with more severe flatfoot accompanied by greater knee degeneration, knee malalignment, knee pain, stiffness and functional impairment (Table 3).

**Table 3:** Participant-level characteristics of the research population based on the severity of flatfoot (*n* = 76).

Variables	Mild flatfoot (*n* = 12 patients)	Moderate flatfoot (*n* = 16 patients)	Severe flatfoot (*n* = 45 patients)	*p*-value*
Subject characteristics				
Age (years)	57.08 ± 11.67	54.13 ± 11.17	57.98 ± 10.41	0.523
Male, *N* (%) / Female, *N* (%)	2 (16.7) / 10 (83.3)	0 / 16 (100)	5 (11.1) / 40 (88.9)	0.29
BMI (kg/m²), Median (IQR)	25.11 (23.98, 27.69)	25.45 (23.26, 28.17)	23.92 (22.59, 28.4)	0.182
Severity of knee OA (K-L grade), *N* (%)	** **	** **	** **	**<0** **.** **001**
K-L 2	**5 (41.7)**	**7 (43.8)**	**0**	** **
K-L 3	**6 (50.0)**	**9 (56.2)**	**30 (66.7)**	** **
K-L 4	**1 (8.3)**	**0**	**15 (33.3)**	** **
FTA (°), Mean **±** SD	−**5.93 ± 1.83**	−**7.52 ± 1.01**	−**8.97 ± 3.31**	**0**.**003**
Pain (WOMAC score), Median (IQR)	**1.79 (1.63, 1.98)**	**1.93 (1.56, 2.16)**	**2.08 (1.93, 2.58)**	**0**.**002**
Stiffness (WOMAC score), Median (IQR)	**1.20 (0.80, 1.20)**	**1.30 (0.80, 1.80)**	**1.60 (1.20, 1.80)**	**0**.**007**
Physical function (WOMAC score), Median (IQR)	**1.38 (1.17, 1.65)**	**1.51 (1.34, 1.89)**	**1.81 (1.68, 2.25)**	**0**.**001**

* *p*-*values from one-way ANOVA test for categorical variables presented as N (%) and normal continuous variables as mean ± SD, Kruskal-Wallis Test for variables presented as median (IQR). Bold values represent statistically significant results*.

The results of the regression analysis in Tables 4, 5 show that having flatfoot was associated with an increased risk of having a higher K-L grade (proportional odds ratio (OR): 20.03; 95% CI, 5.88, 68.27; *p* < 0.001), smaller FTA (beta: −2.96; 95% CI, −4.41, −1.50; *p* < 0.001), higher pain score (beta: 0.47; 95% CI, 0.24, 0.69; *p* < 0.001) and greater dysfunction (beta: 0.25; 95% CI, 0.02, 0.48; *p* = 0.03). The presence of flatfoot was not associated with joint stiffness (*p* = 0.89). The results also demonstrate that more severe flatfoot, from mild to severe, was associated with a higher K-L grade (OR:0.19; 95% CI, 0.08, 0.44; *p* < 0.001), smaller FTA (beta: 1.51; 95% CI, 0.66, 2.35; *p* = 0.001), higher pain score (beta: −0.25; 95% CI, −0.39, −0.11; *p* = 0.001) higher stiffness score (beta: −0.24; 95% CI, −0.38, −0.09; *p* = 0.002) and greater dysfunction (beta: −0.27; 95% CI, −0.41, −0.14; *p* < 0.001).

**Table 4:** Regression analyses on the risk of K-L grade according to the presence of flatfoot and the severity of flatfoot.

	Confirmed presence of flatfoot		Severity of flatfoot	
	OR (95% CI)	*p*-value	OR (95% CI)	*p*-value
Severity of knee OA (K-L grade)^*a*^	**20.03 (5.88, 68.27)**	**<0.001**	**0.19 (0.08, 0.44)**	**<0.001**

^a^ *Ordinal Regression model was fitted. OR: odds ratio; CI, confidence interval. Bold values represent statistically significant results*.

**Table 5:** Regression analyses on the risk of FTA, pain, stiffness and physical function according to the presence of flatfoot and the severity of flatfoot.

	Confirmed presence of flatfoot		Severity of flatfoot	
	Beta (95% CI)	*p*-value	Beta (95% CI)	*p*-value
FTA^*a*^	**−2.96** **(**−**4.41,** −**1.50)**	**<0**.**001**	**1.51** **(0.66, 2.35)**	**0**.**001**
Pain^*a*^	**0.47** (**0.24, 0.69)**	**<0**.**001**	−**0.25** **(−0.39,** −**0.11)**	**0**.**001**
Stiffness^*a*^	−0.02 (−0.29, 0.25)	0.89	−**0.24** **(−0.38,** −**0.09)**	**0**.**002**
Physical function^*a*^	**0.25** (**0.02, 0.48)**	**0**.**03**	−**0.27** **(−0.41,** −**0.14)**	**<0**.**001**

^a^ *Linear regression models were fitted, unless otherwise noted.*

## Discussion

This study assessed the association between flatfoot (presence and severity) and OA-related knee symptoms. The results showed that the presence of flatfoot was significantly associated with more severe OA-related knee symptoms. Additionally, more severe flatfoot was significantly associated with a greater risk of more severe OA symptoms. These findings may be helpful for the treatment and prevention of knee OA in the elderly.

This research investigated 95 patients with knee OA and found that 76.8% had flatfoot. This value is within the range of the prevalence of flatfoot reported in other studies on patients with knee OA when using the same assessment methods (49.5%–95.3%) (2, 10, 16).

It was found that patients with flatfoot had a higher K-L grade, indicating that the presence of flatfoot may be correlated with more severe knee degeneration. This result was consistent with findings reported by Gross et al. (7), in which subjects with flatfoot were significantly more likely to suffer medial cartilage damage than normal subjects (1.4 times). In addition, the K-L grade tended to be higher as the flatfoot degenerated, meaning that having a severe flatfoot tended to be associated with more severe cartilage damage or knee deformities. Therefore, the determination of knee degeneration needs to consider not only the presence of flatfoot but also the severity.

This study showed that femorotibial alignment was not only significantly correlated with the presence of flatfoot but also associated with the severity of flatting. Abnormal foot structure and foot alignment may induce a compensatory mechanism, such as varus knee (24). Foot orthoses are a commonly used conservative treatment that adjusts the FTA by correcting foot posture, therefore balancing the loading on the knee (25). Foot orthoses can reduce the knee adduction moment and improve knee varus by shifting the center of pressure of the foot (26). Once the varus alignment is corrected, high-pressure zones are dispersed, resulting in significant improvements in knee pain and other symptoms of knee OA. This study clarified the association between knee alignment and the presence of flatfoot and found that the FTA was associated with different degrees of flatfoot. Therefore, when assessing joint alignment, it is important to consider how effective the treatment method is for people with different degrees of flatfoot.

Both the presence and the severity of flatfoot were found to be significantly associated with knee pain. Gross et al. (7) found that the patients with flatfoot were at a higher risk of knee pain than those without flatfoot. The relationship between knee pain and flatfoot was examined in a cohort of patients with bilateral flatfoot (10). Knee pain mainly stems from pressure on the knee. Flatfoot not only exhibits a collapse of the arch, but maybe also accompanied by damage of soft tissues in the foot (27) (i.e. the posterior tibial tendon, spring ligament, plantar fascia, and so on). It could be the damage of soft tissues that results in a decline of the shock absorption capacity of the foot (28), and then cause an increase of the force on the knee joint, and knee pain. The results of this current study showed that treating patients individually based on the severity of the flatfoot may better relieve knee pain.

Stiffness of the knee joint was significantly associated with the severity of flatfoot, with higher stiffness scores being reported in the more severe flatfoot group. In a study on patients with bilateral flatfoot, Iijima et al. (10) reported a relationship between flatfoot and knee stiffness. The low number of subjects with unilateral flatfoot in this current study and the limited assessment of knee stiffness with the WOMAC system (29) may limit the interpretation of the association between the presence of flatfoot and knee stiffness.

Guler et al. (2) reported that coexisting flatfoot increased the disability level in patients with knee OA, which supports the finding of this study on the connection between flatfoot and knee function. Symptomatic knee OA is well known to present with functional impairment, and the presence and severity of flatfoot are correlated with the severity of functional impairment. Abnormal lower extremity alignment and knee pain due to a poor arch formation may explain why the patients with flatfoot report more severe physical dysfunction than those without flatfoot.

The relationship between flatfoot and knee OA-induced symptoms in our research suggests that knee OA can be alleviated by taking steps to correct the flatfoot, for example through surgery or orthoses. In addition, this study also documented a significant association between the severity of flatfoot and knee OA, and the severity of flatfoot should be considered when determining a suitable conservative management plan for knee OA.

There are some limitations to this study. First, all participants were diagnosed with knee OA, therefore, there is a high percentage of older (mean age 58 years) and female subjects in the sample pool, who more commonly develop knee OA (30). Thus, the results may not be appropriate for a younger population. Second, the sample size in this study is limited, more research subjects may be more helpful to generalize the results. Third, this study did not consider other influential factors such as ipsilateral foot pain and physical performance when assessing the correlation between flatfoot and knee OA. We cannot preclude the possibility that pain in other joints may contribute to the relationship between flatfoot and knee pain and physical function. Future studies on other possible influential factors are warranted to confirm the negative effect of the severity of flatfoot on knee pain and function. Forth, the results of this study only documented that the presence and the severity of flatfoot were associated with symptoms of knee OA, and the biomechanical mechanism between foot posture and knee OA needs to be further studied in future work. Finally, the cross-sectional design of the study limited our ability to infer a causal relationship between flatfoot and knee OA. The etiology of knee osteoarthritis is multifactorial. The results of this study demonstrated a strong association between flatfoot and knee osteoarthritis, which prompted us to assess the role of flatfoot in knee OA.

## Conclusions

This study systematically investigated whether the presence and the severity of flatfoot are associated with symptoms of knee OA, including knee degeneration, femorotibial alignment, knee pain, stiffness, and dysfunction. The results indicated that both having flatfoot and the severity of flattening were significantly associated with the aforementioned symptoms. For the conservative management of knee OA, both the presence and severity of flatfoot should be carefully considered. These findings provide a base for conservative treatment options for knee OA by modifying foot morphology.

## Data Availability

The raw data supporting the conclusions of this article will be made available by the authors, without undue reservation.
